# Insight into the LED-assisted deposition of platinum nanoparticles on the titania surface: understanding the effect of LEDs

**DOI:** 10.1038/s41598-022-27232-5

**Published:** 2022-12-29

**Authors:** Adam Kubiak, Naisargi Varma, Marek Sikorski

**Affiliations:** Adam Mickiewicz University, Poznań, Uniwersytetu Poznańskiego 8, 61-614 Poznan, Poland

**Keywords:** Materials science, Materials for energy and catalysis, Photocatalysis

## Abstract

This paper proposes a novel LED-assisted deposition of platinum nanoparticles on the titania surface. For the first time, this process was supported by a UV-LED solution. We used two light sources with different wavelengths (λ_max_ = 365 and 395 nm), and power (P = 1, 5, and 10 W) because the photodeposition process based on LEDs has not been defined. The TiO_2_–Pt material was discovered to be nano-crystalline anatase particles with nano-platinum particles deposited on the surface of titanium dioxide. Furthermore, the luminescence intensity decreased when Pt was added to TiO_2_, indicating that charge carrier recombination was reduced. The spectra matching of the photocatalyst and LED reactor was performed for the first time in this work. We proposed a convenient LED reactor that focused light in the range of 350–450 nm, allowing us to effectively use photo-oxidative properties of TiO_2_–Pt materials in the process of removing 4-chlorophenol. In the presented work, the LED light source plays a dual role. They first induce the platinum photodeposition process, before becoming an important component of tailored photoreactors, which is an important innovative aspect of this research.

## Introduction

In today’s world, it is crucial to constantly care for the natural environment to achieve climate neutrality. Even more than ever, we see that the drive to change our economic strategy will achieve climate neutrality and ensure stability in the world's markets^1–3^. As we approach the exhaustion of fossil fuel deposits (resources around the world may last 40 years for oil, 60 years for natural gas, and 200 years for coal), it is critical to use them wisely^4^. However, effective management is required in all areas of technology.

Focusing on the phenomenon of photocatalysis, one must pay close attention to one of the most important aspects of system operation—the photocatalyst. Following Fujishima and Honda’s water-splitting breakthrough in 1972^5^, the photocatalytic properties of certain materials have been used to convert solar energy into chemical energy to remove pollutants and bacteria. Currently, the development of photocatalysts is primarily focused on the formation of advanced materials^6^. This approach can be divided into two main routes—the first focus only on materials active in UV light, while the second aims to use visible light^7^. Among various strategies towards the activity of semiconductors in visible light, the surface modification of wide-bandgap semiconductors (such as TiO_2_^8^, ZnO^9^, Nb_2_O_5_^10^, etc.) with noble metals, including gold^11^, silver^12^, and platinum^13^, has probably been the most popular. Kraeutler and Bard^14^ discovered that platinum could scavenge photogenerated electrons, preventing charge carriers’ from recombining. During, the photodeposition process the metal–semiconductor interface, a Schottky barrier is formed, which slows charge carrier recombination by trapping electrons^15^. When exposed to visible light, TiO_2_ is activated during LSPR decay via electron transfer from metal nanoparticles to the titanium dioxide conduction band^16^. However, according to the scientific literature, a controlled process of precious metal photodeposition is carried out using of conventional UV light sources. As a result, to get the active substance in visible light, we have to use conventional UV light. Alternatively, this process can be carried out in sunlight, but we lose control over material parameters, such as the crystal structure of metallic nanoparticles and morphology, which are critical in terms of subsequent application properties^17^. It should be noted that the photodeposition modification of wide-bang gap photocatalysts extends the absorption band to visible light, without eliminating the absorption band in the UV range. As a result, it has been established that the photodeposition of noble metals can increase photoactivity under UV radiation. This hypothesis was proved by Hu et al.^18^, which showed that the platinum nanoparticles hinder charge carriers’ recombination, resulting in a longer lifetime of photogenerated charge carriers. Similarly, conclusions presented by Kowalska et al.^19^ used the time-resolved microwave to conductivity to show the scavenging of photogenerated electrons by platinum deposited on the titania surface. These results correlated well with the enhanced photocatalytic activity for the oxidative decomposition of phenol and rhodamine B. The authors demonstrated that Pt-modified titanium performed better under both UV and Vis radiation. This demonstrates that Pt-semiconductor materials have two absorption bands, both in the ultraviolet and visible light ranges^20^. Hence, the material’s absorption spectrum should be used as efficiently as possible to ensure higher photocatalytic efficiency. However, the so-called conventional light sources, which are still widely used in photocatalysis, cannot be used for this because both the xenon and mercury lamps are discharge lamps, and the spectrum cannot be adjusted to the material. LED light sources, which allow for wavelength control, are an option here^21^. The diamond (235 nm) and III-nitride semiconductors such as indium gallium nitride (InGaN; 365–410 nm), boron nitride (BN; 215 nm), aluminum nitride (AlN; 210 nm) are used in the LED solutions^22^. The central emitting wavelength of GaN is 365 nm, and longer UV wavelengths use InGaN. The InGaN-based diodes emit UV-A light, whereas AlGaN and AlInGaN-based diodes are characterized by the primary UV-B and UV-C output wavelengths depending on Al, Ga, and the ratio^23^. By using Al instead of In in the nitrides, shorter UV wavelengths can be produced^24^. This shows how a variable-wavelength light source can be obtained using commercial LEDs^25^. However, so far in the field of photocatalysis, no attempt has been made to match LED light sources with a photocatalyst.

To meet the actual expectations, this work proposed a novel LED-assisted deposition of platinum nanoparticles on the titania surface. The main element of the novelty is the use of LED light sources in the photodeposition process, which, to the best of our knowledge, is not common in the available scientific literature. To understand the influence of LEDs on the deposition process, we used two light sources with different wavelengths (λ_max_ = 365 and 395 nm) and power (P = 1, 5, and 10 W). What's more, for the first time, the spectra matching of the photocatalyst and LED reactor was carried out. We proposed a convenient LED reactor focusing light in the range of 350–450 nm, which allowed for the effective use of photo-oxidative properties of TiO_2_–Pt materials in the process of removing 4-chlorophenol.

## Materials and method

### Materials

Titanium tetrachloride (97%), urea (p.a.), chloroplatinic acid hydrate (≥ 99.9%), and 4-chlorophenol (99%) were purchased from Sigma-Aldrich. All reagents were of analytical grade and used without any further purification. The water used in the experiments had been deionized.

### Synthesis of TiO_2_–Pt materials

The microwave technique was used to prepare the reference titania. The titanium(IV) chloride was used as the precursor, and the mentioned solution was prepared in distilled water in an ice-water bath using a previously reported procedure^26^. The concentration of titanium(IV) chloride was adjusted to 1%. Next, 5 g of urea was added to the 100 cm^3^ TiCl_4_ solution. Subsequently, the solution was transferred to a microwave reactor (CEM, Discover 2.0, USA) and heated with a maximum power of 300 W until it reached 200 °C, then held for 1 min. The obtained titanium dioxide (TiO_2_ NPs) was filtered, washed, and dried at 60 °C for 6 h.

In the next stage, TiO_2_–Pt materials were prepared by photodeposition. For this purpose, 1 g of TiO_2_ was dispersed in water: methanol solution (1:1 v/v), and then a specified amount of chloroplatinic acid hydrate was added to obtain 1%wt. of Pt. After that, the vessel was sealed and purged with nitrogen for 30 min. The suspension was then irradiated to monochromatic LED light (λ_max_ = 365 or 395 nm) with variable power (P = 1, 5, or 10 W) for 1 h. After the process, a color change was observed from orange (resulting from the addition of the platinum precursor) to gray, confirming the effective reduction of Pt. The samples were marked with the formula defining the deposition parameters: (power)_(wavelength) hence e.g.: 1W_365 nm, 5W_365 nm, 10W_365 nm, etc.

### Characterization of fabricated materials

The X-ray diffraction method (XRD) was used to determine the crystalline structure. XRD analysis was performed with a D8 Advance diffractometer (Bruker, Germany) operating with Cu Kα radiation (α = 1.5418 Å), Ni filtered. The patterns were obtained in step-scanning mode (Δ2θ = 0.05°) over an angular range of 20–80°. The analysis was based on the International Centre for Diffraction Data (ICDD) database.

The high-resolution transmission electron microscopic (HR-TEM) measurements were performed with a Hitachi HT7700 microscope (Hitachi, Japan) operating at 100 kV. Preparation of the sample for measurements consisted in dispersed a small quantity of the sample in 2 cm^3^ of deionized water with the use of ultrasound. Then, 1 μl of the solution was applied to a nickel mesh covered with a carbon film.

The BET surface area, pore volume, and pore size were determined using a 3FLEX surface characterization analyzer (Micromeritics Instrument Co., USA) by the Brunauer–Emmett–Teller (BET) method based on low-temperature N_2_ sorption. The surface area was determined by the multipoint BET method using adsorption data in a relative pressure (*p*/*p*_0_) range of 0.05–0.30.

The morphology of the fabricated materials was determined using a transmission electron microscope working in high contrast mode (Hitachi HT7700, Hitachi, Japan). The maps of titanium and platinum elements were performed using the mentioned microscope operating in the STEM mode with a system to the energy dispersive X-ray microanalysis (Thermo Scientific, USA).

Atomic absorption spectroscopy ContrAA300 (Analytik Jena, Germany) was applied to determine metal ions concentrations in the aqueous solutions at the wavelength 265.9 nm. The calibration curve y = (0.0000627 + 0.0015604x)/(1 + 0.0017031x) was used to determine the concentration of Pt (IV).

The diffuse reflectance spectroscopy (DRS) was carried out using a Thermo Scientific Evolution 20 (Thermo Scientific, USA) spectrophotometer equipped with a PIN-757 integrating sphere. The bandgap energies of the obtained samples were calculated based on the Kubelka–Munk function:

1 $$F\left(R\right)=\frac{{\left(1-R\right)}^{2}}{2R}$$

where R is reflectance, which is proportional to the absorption of radiation, by plotting:

2 $$F{\left(R\right)}^{0.5}{E}_{ph}^{0.5}$$

where E_ph_ means the photon energy.

The photoluminescence (PL) measurements were carried out using a spectrofluorometer (Fluorolog version-3 Horiba, Japan) with a 450 W high-pressure xenon arc lamp as an excitation source. The photoluminescence excitation (λ = 350 nm) and emission spectra were acquired at room temperature at a spectral resolution of 1 nm at a slit width of 1 mm.

### Photocatalytic test

#### Light source

**Figure 1:**
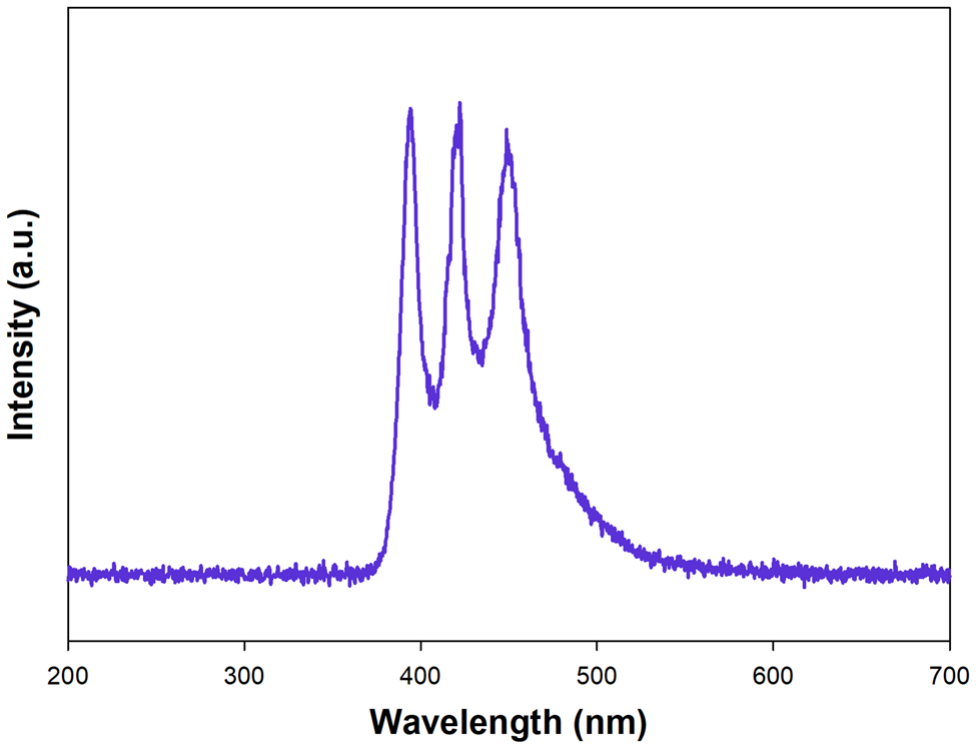
The spectrum of matched LED light source used for photooxidation processes.

The spectra-matched LED light source was based on the chip-on-board diodes with an λ_max_ = 395, 425, and 450 nm. Single 10 W COB LEDs (Bridgelux, USA) are placed on the aluminum heat sink and was connected with a dimmer that allows you to conveniently change the power of the light source in the range of 1–10 W for each LED. Next, the LED system was connected to the ballast (Mean Well, Taiwan). The working power of 20 W was confirmed using a GB202 wattmeter (GreenBlue, China). The spectrum of the designed LED photoreactor is shown in Fig. 1.

#### Photo-oxidation test

The 100 cm^3^ of the 4-chlorophenol (20 mg/l) and 100 mg of the TiO_2_–Pt photocatalyst were introduced into the LED reactor. The resulting suspension was homogenized using a magnetic stirrer (IKA Werke GmbH, Germany) in darkness (30 min) to establish adsorption/desorption equilibrium. Next, the matched LED solution was switched on, and the reaction mixture was irradiated. Every 20 min (up to 120 min, then stopped the irradiation), 3 cm^3^ of the suspension was collected and filtered through a syringe filter (Macherey–Nagel, Germany). The filtrate was analyzed using a UV–Vis spectrophotometer (UV-2550, Shimadzu, Japan) in the 200–700 nm wavelength range, using the demineralized water spectrum as a baseline. The maximum absorbance of pollution at wavenumber 280 nm was observed. The photocatalytic activity of the samples was determined by applying a calibration curve method with the formula y = 0.01x + 0.277, where x was the 4-chlorophenol concentration, and y was the maximum absorbance value.

## Results and discussion

### Crystal and textural properties

**Figure 2:**
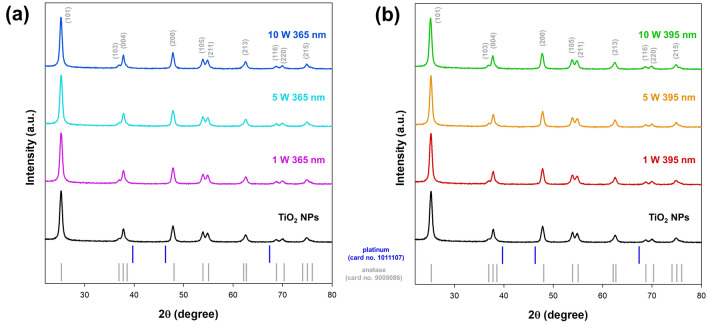
The XRD patterns for samples series: (**a**) 365 nm and (**b**) 395 nm.

To verify the effect of the LED-induced Pt deposition process on the titania surface, an XRD analysis was performed. The collected data is presented in Fig. 2.

**Figure 3:**
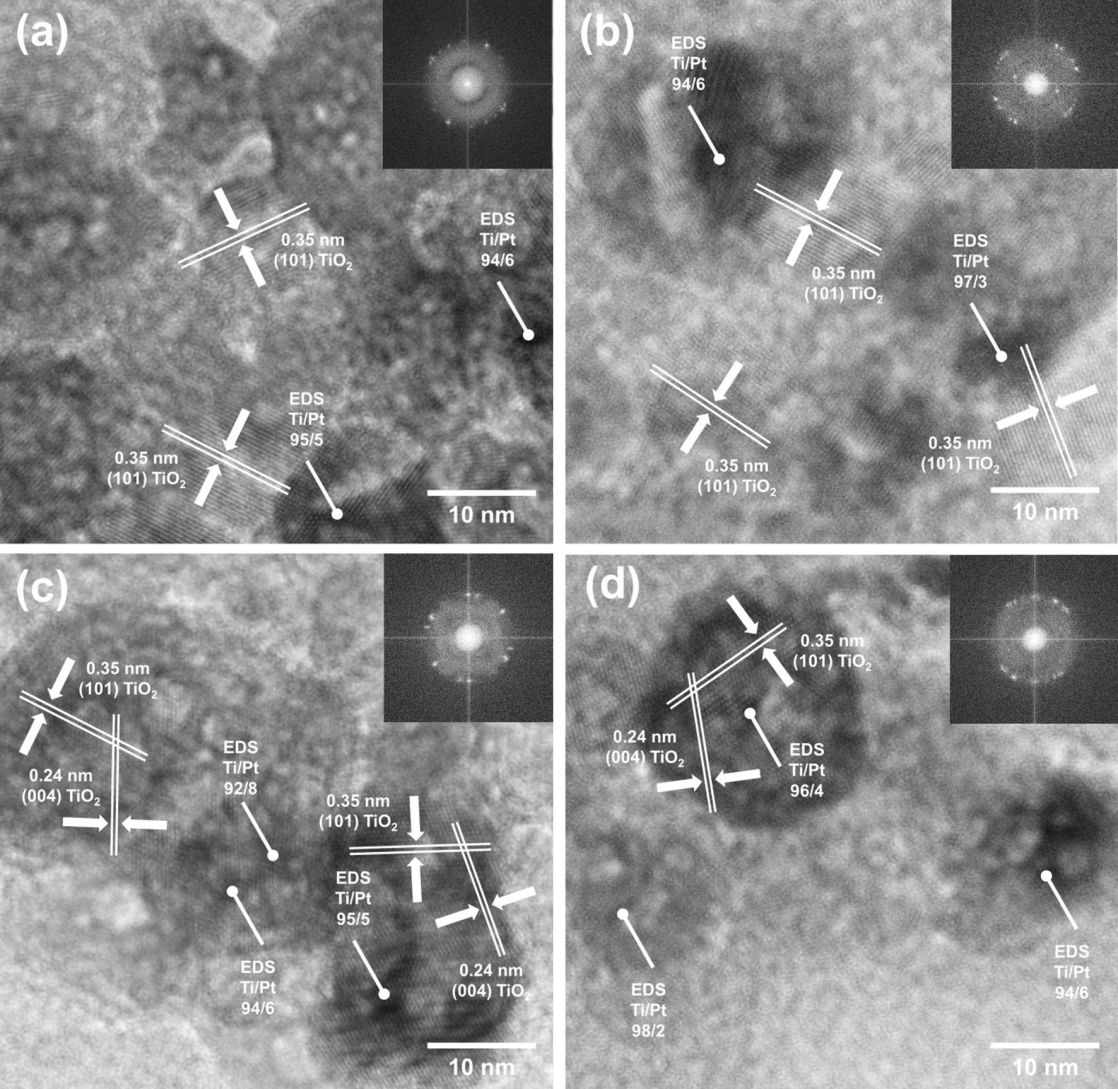
The HR-TEM and FFT images for selected samples: (**a**) 1 W 365 nm, (**b**) 10 W 365 nm, (**c**) 1 W 395 nm, and (**d**) 10 W 395 nm.

The XRD patterns of TiO_2_ NPs show characteristic peaks at 2θ values of 25.28, 36.9, 37.8, 47.9, 53.8, 55, 62.6, 68.7, 70, and 75.05 which are strictly related to the anatase phase (card no. 9009086)^27^. The average crystallite size for the mentioned material was 9.8 nm. No apparent differences were observed in the XRD patterns for materials containing Pt nanoparticles. Regardless of the LED light source and its power, the obtained TiO_2_–Pt materials had a similar crystallite size in the range of 9.6–9.8 nm for the anatase phase. The presence of platinum was not approved by XRD analysis (no peaks for Pt particles) due to their low content (1 wt%) and nanometric size. No other crystalline phases were identified in the patterns, which indicated the crystal purity of the obtained materials. Similar conclusions were also reported by Bielan et al.^28^, who pointed out that the low content of noble metals was not observed in the XRD patterns. Therefore, the HR-TEM analysis was carried out to comprehensively characterize crystal structure. The obtained results are presented in Fig. 3.

The presented high-resolution images confirmed that the obtained materials are nano-crystalline particles of anatase. The samples from the 365 nm series are correctly oriented with respect to the incident electron beam, showing lattice fringes spaced 0.35 nm apart (d_101_ = 0.3516 nm)^29^. On the other hand, for the 395 nm series samples, an additional 0.24 nm lattice fringes corresponding to the (004) TiO_2_ plane (d_004_ = 0.2430 nm) were observed^30^. The absence of the above-mentioned lattice fringes for the 365 nm sample series may indicate that {001} facets were effectively covered with Pt nanoparticles and therefore could not be observed in high-resolution images. The available scientific literature confirms the effect of facets on the photodeposition of noble metal nanoparticles^16,31^. Ohno et al.^32^ and Wang et al.^33^ confirmed that facets on TiO_2_ crystals play a crucial role in the enhancement of the photodeposition of noble nanoparticles. However, the influence of the light source on the photodeposition of Pt nanoparticles on the facets of TiO_2_ crystals has not been described so far. It seems that the higher energy of the applied UV radiation, λ_max_ = 365 nm, allows for the coating of the high-energy {001} facets of TiO_2_ in the first place. Moreover, it was found that the platinum nanoparticles are well dispersed on the nano-TiO_2_ surface. They are visible as dark spots on HR-TEM images, which was confirmed by EDS point analysis. Similar observations were also described by Wysocka et al.^15^.

**Figure 4:**
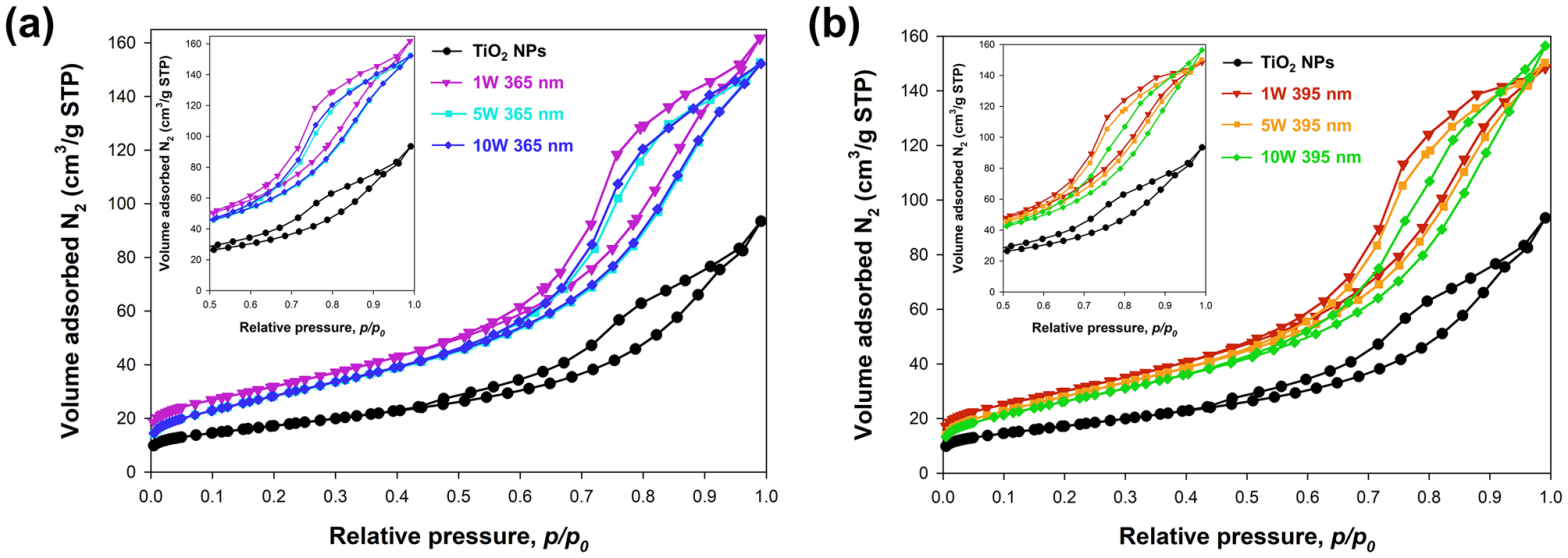
The adsorption–desorption N_2_ isotherm for samples series: (**a**) 365 nm and (**b**) 395 nm.

In addition to crystallography, another of the key parameters of describing the surface of nanomaterials is surface area development. For this purpose, low-temperature nitrogen sorption was carried out. The obtained nitrogen adsorption–desorption isotherms are shown in Fig. 4, while the values of the BET surface area, volume, and pore diameter are presented in Table 1.

**Table 1:** Low-temperature N_2_ sorption results including BET surface area, pore volume, and diameter for synthesized TiO_2_ nanomaterials.

Sample	BET surface area		
	A_BET_ (m^2^/g)	V_p_ (cm^3^/g)	S_p_ (Å)
TiO_2_ NPs	62	0.144	9.2
1 W 365 nm	115	0.244	7.9
5 W 365 nm	108	0.248	8.0
10 W 365 nm	106	0.253	8.2
1 W 395 nm	110	0.234	8.0
5 W 395 nm	105	0.240	8.2
10 W 395 nm	100	0.262	8.4

In the case of the TiO_2_ NPs sample the N_2_ isotherms showed characteristic features of type IV, with reversible mono- and multilayer adsorption in the lower range of *p/p*_*0*_, followed by the type H1 hysteresis loop^34^. This type is often associated with porous materials consisting of agglomerates or compacts of approximately uniform spheres in a fairly regular array, and hence have narrow distributions of pore size. Detailed sorption measurements found that the BET surface area was 62 m^2^/g, while the volume and diameter of pores were 0.144 cm^3^/g, and 9.2 nm, respectively.

Regardless of the light source used, the photodeposition of platinum nanoparticles increases the BET surface area in the range of 100–110 m^2^/g. The observed increase in the BET surface area for samples after the photodeposition process was also described by Abdennouri et al.^35^. The authors indicate that the presence of platinum nanoparticles on the TiO_2_ surface leads to an increase in surface area development. The analysis of the obtained N_2_ isotherms showed that the increase in the power of the light source influences the porous structure, which is observed by the narrowing of the hysteresis loop. This change is visible for the 395 nm sample series, where the hysteresis loop type changes from H1 (1 W 395 nm) to H3 (10 W 395 nm)^34^. The Type H3 loop is observed with aggregates of plate-like particles giving rise to slit-shaped pores. This proves that the LED-induced Pt photodeposition process leads to a change in the shape of the pores from cylindrical to wedge-shaped^34^. The mentioned change in the porous structure is also observed in detailed sorption measurements. In the case of the 1 W 395 nm sample, the volume, and pores diameter were 0.221 cm^3^/g and 6.5 nm, respectively. On the other hand, for the 10 W 395 nm sample, an increase in the volume and the pore diameter to 0.262 cm^3^/g and 8.4 nm was noted. Furthermore, it was found that the observed change in the shape of the pores result in a decrease in BET surface area. On the other hand, an increase in total pore volume and diameter was noted. Nevertheless, it is worth emphasizing that the influence of the light source on the development of surface area TiO_2_–Pt nanoparticles has not been described so far.

### Morphology and surface composition

**Figure 5:**
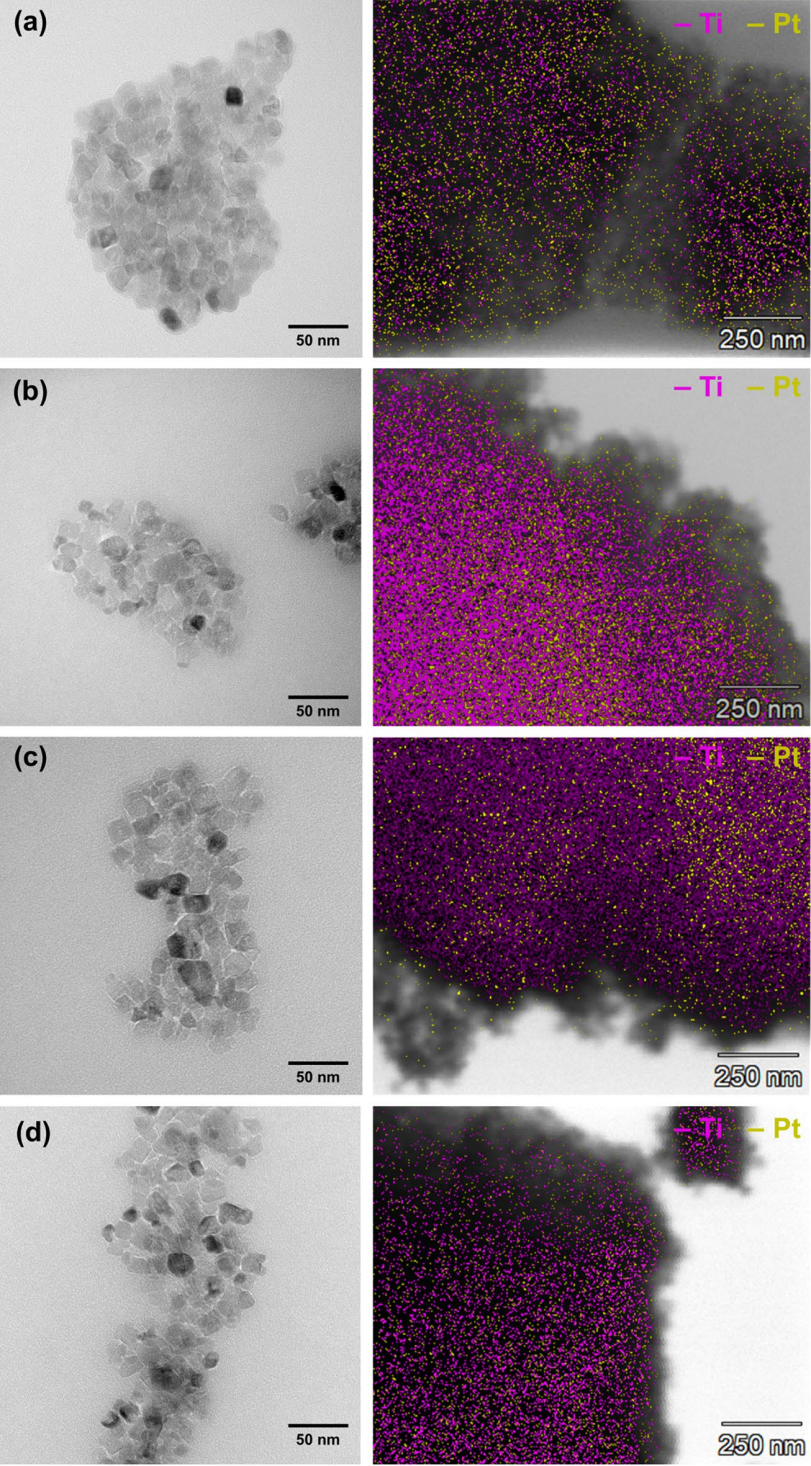
The TEM and EDS map (pink color means titanium; yellow color means platinum) images for selected samples: (**a**) 1 W 365 nm, (**b**) 10 W 365 nm, (**c**) 1 W 395 nm, and (**d**) 10 W 395 nm.

Particle size control, particularly on the nanoscale, is fundamental for materials applications, and transmission electron microscopy (Fig. 5) was carried out to characterize this important parameter. Moreover, to confirm the effectiveness of the photodeposition process, EDS mapping was carried out to determine the distribution of platinum in the obtained materials.

Regardless of the analyzed TiO_2_–Pt material, the presence of octahedral and cubic particles corresponding to the anatase phase was found. Their size was close to the determined average crystallite size, which confirms that the obtained materials are aggregates of nanocrystalline particles. Since platinum nanoparticles obtained by photodeposition have a size of 5–8 nm, EDS mapping was carried out to determine their distribution in the obtained materials. Analyzing the distribution of titanium (pink color) and platinum (yellow color) indicated it was found that platinum is distributed homogeneously on the surface of analyzed TiO_2_–Pt materials. The Pt nanoparticles are observed as a thin layer at the edge of the TiO_2_ nanocrystalline particles, particularly well visible for the 1 W 365 nm (Fig. 5a) and 1 W 395 nm (Fig. 5c) samples. Moreover, according to the available literature, platinum particles obtained by photodeposition can also be observed in the form of dark areas on single crystals of the base material—which is also observed in our research^36–38^. Nevertheless, the Pt content determined based on EDXRF analysis was close to the assumed theoretical values and ranged from 0.8 to 1.1% by weight. Furthermore, to confirm the high efficiency of the LED-induced Pt photodeposition process proposed by us, we conducted AAS measurements. The obtained results indicate that almost all content of platinum has been deposited on the surface of titanium dioxide. Regardless of the tested material, the concentration of platinum in the solution after the photodeposition process was in the range of 0.07905–0.08196 Pt mg/L.

### Optical properties

**Figure 6:**
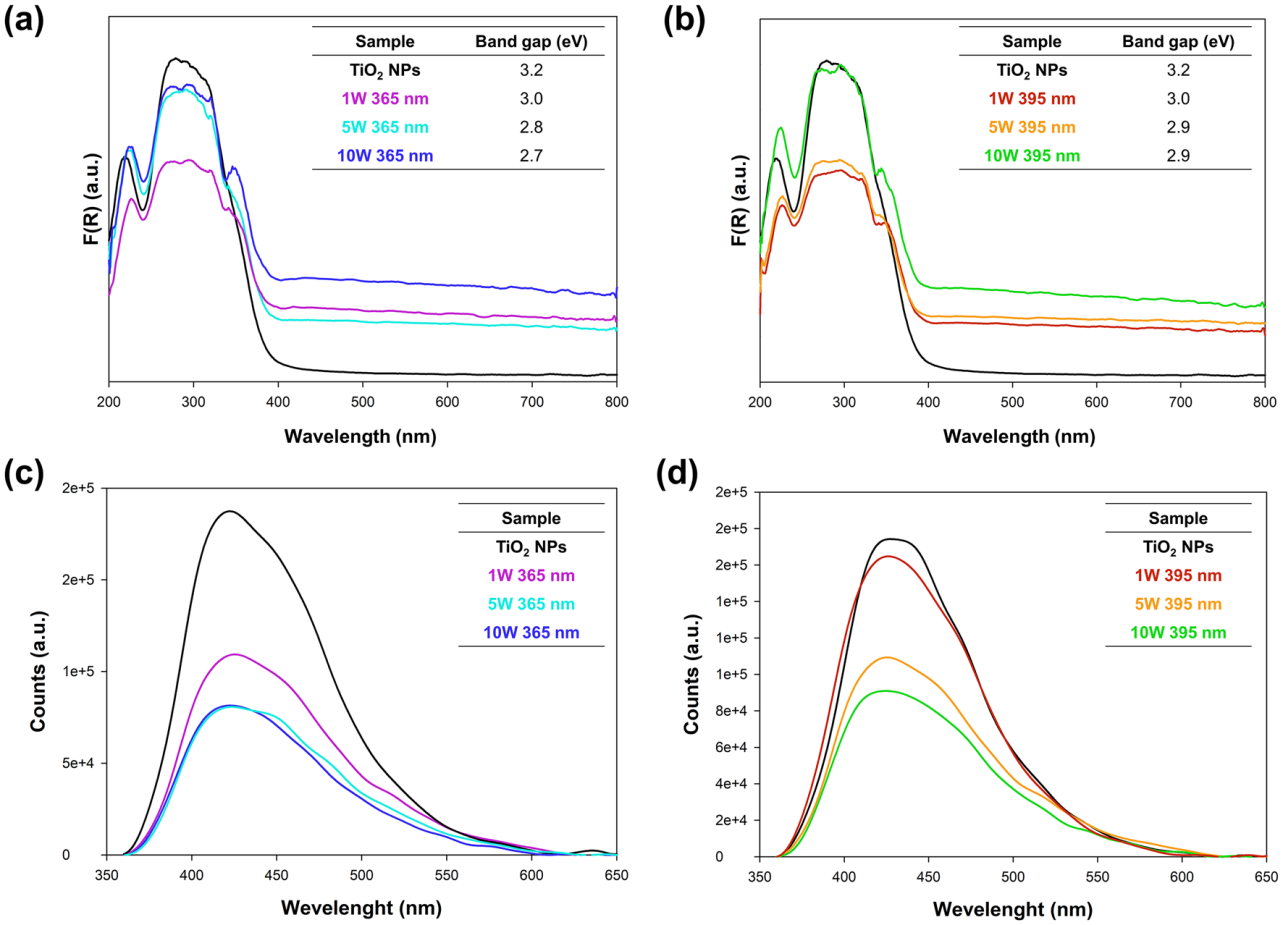
The (**a**,**b**) DRS and (**c**,**d**) fluorescence spectra for sample from (**a**,**c**) 365 nm and (**b**,**d**) 395 nm series.

Taking into account the presence of platinum nanoparticles in the structure of the synthesized materials, which could modify optical properties, diffuse reflection spectroscopy (DRS) and photoluminescence spectroscopy (PL) were performed (Fig. 6).

First of all, it should be noted that the platinum nanoparticles modify the optical properties and thus the energy of the band gap (Fig. 6a,b). This proves that the modification does not only occur on the surface but also interacts with the structure of TiO_2_^39^. The use of the lowest power of the LEDs leads to a reduction of the borne band energy to 3.0 eV, compared to 3.2 eV for the reference sample (TiO_2_ NPs). Additionally, there is a widening of the absorption band in the range of 390–420 nm. A further increase in the power of the light source resulted in a reduction of the band gap energy—2.8 eV for 5 W 365 nm and 2.9 eV for 396 nm, respectively. However, only by using the maximum power of the LEDs (10 W), a further extension of the absorption spectrum was noted—for the 365 nm series to 450 nm, while for the 395 nm series to 430 nm. The change in absorption spectra for analyzed samples is associated with the presence of Localized Surface Plasmon Resonance (LSPR) peaks for Pt^40^. Platinum surface plasmon resonance was observed at the wavelength of about 410–420 nm. This confirms the electron transfer between Pt nanoparticles and the valence band of titanium dioxide^15^.

In the next step, of the comprehensive analysis of synthesized TiO_2_–Pt materials, exploited photoluminescence spectroscopy (Fig. 6c,d), which provides information on surface processes involving the recombination of photogenerated charge carriers. Photoluminescence can be generated during the recombination of the photogenerated carriers on titanium dioxide^41^. We observed a luminescence band near ∼ 450 nm characteristic of pure TiO_2_. In the literature TiO_2_, the photoluminescence spectrum has two main emission peaks that appear at about 396 and 462 nm wavelengths, which are equivalent to 3.13 and 2.68 eV, respectively^42^. However, broad luminescence bands are also known, as being the sum of these narrow peaks, as reported by Chang et al.^43^. What is important, is these peaks are ascribed to the emission of bandgap transition with the energy of light approximately equal to the bandgap energy of anatase (387.5 nm) and emission signal originating from the charge-transfer transition from Ti^3+^ to oxygen anions in a TiO_6_^8−^ complex. Regardless of LEDs power when Pt was added to TiO_2_, the intensity of the luminescence band at ∼450 nm decreased, suggesting a reduced charge carrier recombination^44^. This can be explained by the migration of excited electrons from TiO_2_ to the Pt nanoparticle, preventing electron–hole recombination^45^. Our result suggests that the higher power of LEDs is more efficient to extract the electrons from TiO_2_. Apart from the significant decrease in the luminescence intensity band, it should be noted that the position of the peaks has changed. For the reference TiO_2_ sample, the peak maximum is 430 nm. On the other hand, for TiO_2_–Pt materials shifts the luminescence maximum towards lower wavelengths—421 nm. The mentioned effects are attributed to the efficient charge separation, which prevents the direct recombination of electrons and holes.

### Photocatalytic activity

**Figure 7:**
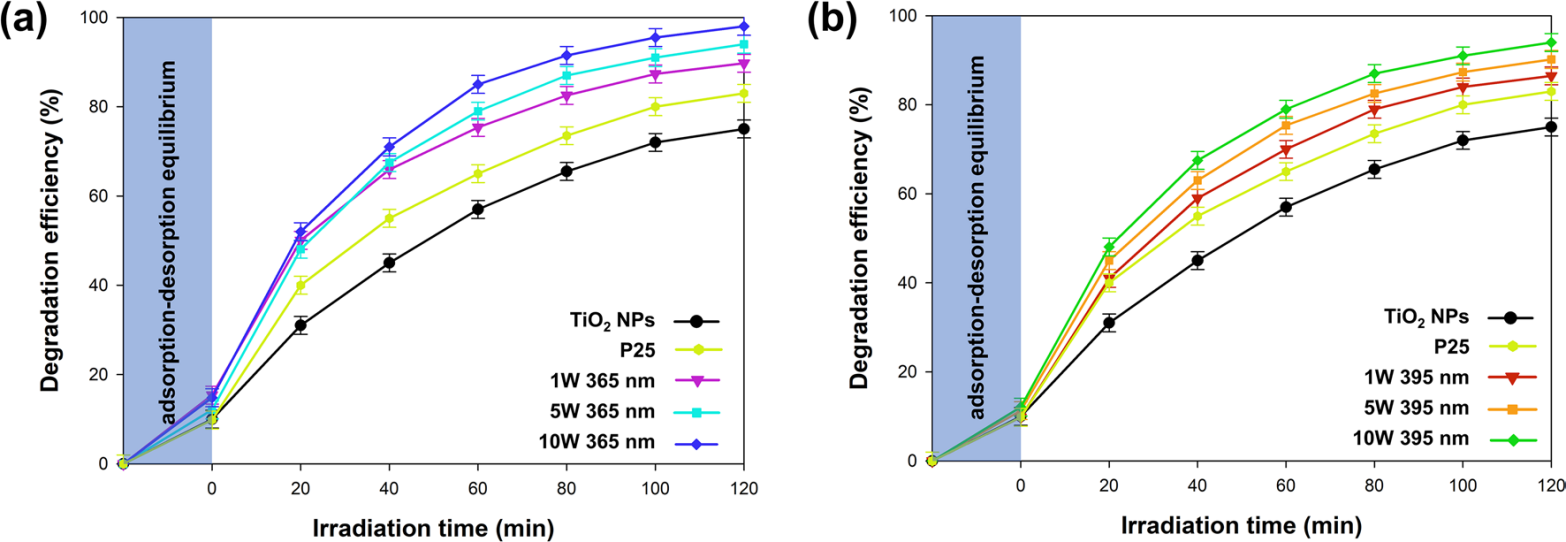
The photo-oxidation results of samples from (**a**) 365 nm and (**b**) 395 nm series.

The 4-chlorophenol is used extensively in various industries such as petroleum refining, gas and coke production, and varnish formulation^46,47^. The permissible limit of mentioned phenol derivative is 1 mg/l for industrial effluents to be discharged into inland surface waters (IS: 2490-1974) and 5 mg/l for discharge into public sewers (IS: 3306–1974)^48,49^. However, the concentration in industrial wastes ranges from 50 to 2000 mg/l^50,51^. Therefore 4-chlorophenol (initial concentration 20 mg/l) has been selected as a model organic pollutant due to its prevalence and significant threat to the natural environment. The collected photo-oxidation curves are shown in Fig. 7.

Regardless of the light source used at the photodeposition stage, the obtained TiO_2_–Pt materials showed higher efficiency of 4-chlorophenol removal compared to the reference TiO_2_ sample and the commercial P25 sample. The higher photo-oxidative activity of P25 was due to the difference in phase composition compared to TiO_2_ NPs. The titanium dioxide we obtained contained only the anatase phase, hence it only absorbed UV radiation. On the other hand, P25 is a mixture of anatase and rutile^52^, therefore it can also absorb visible light. In the case of both analyzed series (365 nm and 395 nm), the highest phenol removal efficiency was obtained for materials obtained with the maximum power of LEDs for Pt photodeposition, 98% for 365 nm and 95% for 395 nm. Reducing the power of the light source used during photodeposition resulted in a reduction of the subsequent photo-oxidative activity. For samples obtained with the lowest power of light sources, the results were 88% for 365 nm and 86% for 395 nm. Hence, it was proved that the power of the light source used at the photodeposition stage has a key impact on the degradation efficiency of the tested pollutants, in this case, 4-chlorophenol. However, it should be noted that an excessive increase in the power of the light source may also have negative effects, which we have described elsewhere^53^. One of the negative effects is, among others the possibility of overheating the sample and thus an increase in evaporation^54^. Keeping in mind that the photodeposition processes are carried out in sealed vessels, increased evaporation will lead to an increase in pressure, and extreme cases may lead to the destruction of the reactor. Therefore, it can be concluded that the 10 W used by us allows for obtaining an effective photocatalyst while avoiding the negative effects of the excessive power of the light source. Moreover, based on the obtained results, the influence of the wavelength used in the photodeposition process on the efficiency of 4-chlorophenol removal was observed. Based on our results, it was shown that materials formed using LEDs with a wavelength of 365 nm showed a higher photooxidation efficiency. These results are also consistent with the comprehensive physicochemical characteristics carried out. Changing the wavelength of the LED light source at the photodeposition stage determined, among other things, the optical properties—the energy of the band gap and photoluminescence. The higher efficiency of photodeposition at 365 nm was also confirmed based on the obtained TEM images, where it was proved that for the samples from the 365 nm series, no {001} facets, which were probably covered with platinum nanoparticles, were not observed. As the basis for effective Pt photodeposition with the use of a 365 nm LED light source, it is necessary to point out the probably more efficient titanium dioxide excitation process^55^. This allowed for the efficient generation of holes constituting the basic factor determining the photodeposition of platinum. It should be noted, however, that our results are inconsistent with the reports presented by Zhang et al.^56^. The authors noted that the photodeposition of platinum on the Bi_2_WO_6_ catalyst is most effective when using an LED excitation source with a wavelength of 450 nm. The observed differences may be related primarily to the base material, in our research, we used nanocrystalline anatase obtained in situ, which absorbs only UV radiation. In the case of Bi_2_WO_6_, it also shows the absorption of visible light. In addition, attention should be paid to the differences in the LED solutions used. Hence, it is necessary to better understand the influence of the excitation wavelength on the photoreduction process to fine-tune the microstructures and crystallinity of the cocatalyst to improve the physical properties of the photocatalysts^57^.

Based on the available literature, intermediates such as phenol, oxalic acid, and acetic acid were observed in 4-chlorophenol photooxidation. In this case, photodegradation begins with an attack of the reactive oxygen species on the para position of the aromatic ring and subsequent loss of chlorine, causing the formation of phenol and hydroquinone. In further photo-oxidation, hydroquinone is converted to p-benzoquinone and could be decomposed to the observed oxalic acid and acetic acid^58^. This indicates that the high concentration of reactive oxygen species, including superoxide and hydroxyl radicals, is crucial for the rapid course of photooxidation. On the other hand, the formation of hydroxyl radicals is constant for a given amount of the catalyst. It should be noted that Alimoradzadeh et al.^59^, found the reaction rate constant *(k)* of the photocatalytic process, decreased with increasing the initial concentration of 4-chlorophenol. The main reason is that the available OH radicals are insufficient for 4-CP degradation at higher concentrations. Therefore, it should be expected that the increase in the initial concentration of 4-chlorophenol will cause a decrease in the photo-oxidation efficiency obtained for TiO_2_–Pt materials. The higher the concentration of 4-chlorophenol, the greater the concentration of intermediate products that compete for reaction with hydroxyl radicals generated by photogenerated holes trapped on the surface^60^.

Based on the available scientific literature about the photooxidation of 4-chlorophenol, various semiconductors are used including TiO_2_, ZrO_2_, ZnO, and SnO_2_. In our earlier work^61^, we presented a combination of ZnO nanorods and a LED light source (λ_max_ = 65 nm), which allowed us to obtain nearly 90% degradation efficiency after 3 h of irradiation. Ghosh et al.^62^, described the photooxidation of 4-chlorophenol using the Coumarin (C-343) sensitized TiO_2_, which results in nearly 90% efficiency after 6 h. In another paper, Zhu et al.^63^ fabricated the plasmon Ag/MFe_2_O_4_ photocatalysts. The highest efficiency of removing 4-chlorophenol was demonstrated by Ag/CoFe_2_O_4_ material, which after 2 h achieved 90% photooxidation efficiency. High photocatalytic activity toward 4-chlorophenol degradation was demonstrated by Castillo-Rodriguez et al.^64^, who synthesized the Zr_x_O_y_-Bi_2_O_2_(CO_3_) composite. This material had a near 95% photodegradation efficiency after 6 h, under UV-C (254 nm) irradiation. Whereas Yang et al.^65^, presented the fabrication of a three-dimensional hierarchical BiOBr/Bi_2_O_4_ composite with excellent visible light photodegradation performance for 4-chlorophenol.

Nevertheless, the use of a suitable LED light source, in combination with the TiO_2_–Pt photocatalyst, allowed us to obtain much better results in the removal of 4-chlorophenol compared to the current state of knowledge. In addition, we would like to point out that scientists used conventional high-power mercury or xenon lamps (125–500 W) in most of the mentioned works. These considerations demonstrate that the presented approach to spectra-matching of photocatalysts and LED solutions can be a new and unique strategy to develop novel high-efficiency photocatalytic systems for the removal of organic impurities.

## Conclusions

We have described a novel LED-assisted deposition of platinum nanoparticles on the titania surface. The proposed methodology allows obtaining the nano-crystalline particles of anatase with nano-platinum particles deposited on the surface of titanium dioxide. Based on the HR-TEM images found that the 365 nm sample series is absent of 0.24 nm lattice fringes, unlike the 395 nm series. This indicates that {001} facets were more effectively covered with Pt nanoparticles for excitation wavelength λ_max_ = 365 nm. The analysis of the textural properties showed that the increase in the power of the LED light source (λ_max_ = 365 nm) influences the porous structure, which is observed by the narrowing of the hysteresis loop. This proves that the excitation wavelength of the Pt photodeposition process influences the porous structure, and leads to changes in the shape from cylindrical to wedge-shaped. The optical properties indicated that the platinum nanoparticles modify the optical properties and thus the energy of the band gap. This proves that the modification does not only occur on the surface but also interacts with the structure of TiO_2_.

The efficacy of TiO_2_–Pt materials as catalysts in the photodegradation of the 4-chlorophenol was evaluated using a spectra-matched LED photoreactor. The efficiency of the catalyst turned out to be dependent on the power of the light source used in the platinum photodeposition stage. Since the Pt photoreduction process was the most effective with the use of LEDs with λ_max_ = 365 nm and P = 10 W, which was confirmed by the physicochemical analyzes carried out, this material also showed the highest ability to remove 4-chlorophenol. Nevertheless, all the fabricated TiO_2_–Pt materials had higher photo-oxidation efficiency compared to the reference TiO_2_ NPs and the commercial P25 sample. The presented approach to spectra-matching of photocatalysts and LED solution proves to be a new and unique strategy to develop novel high-efficiency photocatalytic systems.

## Data Availability

The data that support the findings of this research are available from the corresponding author upon reasonable request.
